# Mosaic Form of von Hippel–Lindau Syndrome: Case Report and Literature Review

**DOI:** 10.3390/ijms26062751

**Published:** 2025-03-19

**Authors:** Dmitry S. Mikhaylenko, Natalya B. Kuryakova, Anna V. Efremova, Ilya V. Volodin, Sergey I. Kutsev, Dmitry V. Zaletaev, Vladimir V. Strelnikov

**Affiliations:** 1 Research Centre for Medical Genetics, 115522 Moscow, Russiavstrel@list.ru (V.V.S.); 2 Department of Translational Medicine and Biotechnology, Sechenov University, 119991 Moscow, Russia

**Keywords:** von Hippel–Lindau syndrome, mosaic mutation, familial cancer counseling, next-generation sequencing, *VHL* gene

## Abstract

von Hippel–Lindau syndrome (VHLS) is a hereditary cancer syndrome with CNS hemangioblastomas, clear cell renal carcinoma, pheochromocytoma, retinal angiomas, and a number of other manifestations. VHLS is caused by a mutation in the *VHL* gene and is inherited in an autosomal dominant manner. However, some cases of VHLS develop de novo, and among them, there are rare patients with a mosaic form of the disease. Genetic testing in mosaic patients is prone to false-negative results due to the low copy number of a mutant allele in DNA isolated from the blood. We describe a case of molecular genetic diagnostics of VHLS in a 39-year-old patient using various methods, including mutation analysis in asynchronous primary tumors and repeated DNA analysis from blood using NGS with high coverage for the mutant position. As a result, the patient was diagnosed with a mosaic form of VHLS caused by the variant c.481C>T (p.Arg161Ter), the proportion of which in the blood DNA was 2%. We also summarized the literature data on the mosaic form of VHLS: the severity of clinical manifestations, the features of differential diagnostics of VHLS with a negative result of routine molecular genetic *VHL* testing, and specific options of active surveillance and treatment for mutation carriers.

## 1. Introduction

von Hippel–Lindau syndrome (VHLS) is a hereditary cancer syndrome. Clinically, this syndrome is manifested by clear cell renal carcinoma (CCRC) and CNS hemangioblastomas (the most common manifestations); approximately a third of patients develop pheochromocytoma. Retinal angiomas, pancreatic neuroendocrine tumors, cystadenomas of the epididymis and broad ligament of the uterus, and endolymphatic sac tumors are observed in VHLS less frequently. Non-oncological clinical manifestations include renal and pancreatic cysts. VHLS is caused by germline mutation in the *VHL* tumor suppressor gene, which is localized on 3p and is inactivated in the tumor according to the Knudson two-hit model: one allele harbors germline “loss of function” mutation and the second allele acquires alteration in tumor cells due to somatic point mutation, large deletion (“loss of heterozygosity”), or promoter methylation. The syndrome is inherited in an autosomal dominant manner with high penetrance. The VHLS frequency varies within the range of 1:27-91 000 newborns in previously published articles, with most typical estimations of around 1:36 000; however, the frequency of individuals with pathogenic variants calculated in November 2023 using the gnomAD version v2.1.1 database was 1:4.4-8.9 000 in the Caucasian population, which indicates underestimation of VHLS frequency in the past [1,2,3]. Thus, VHLS could be responsible for up to 5% of CCRC cases according to epidemiological estimates, especially in young patients. The average manifestation age for CCRC is 40–45 years, and the lifetime risk is about 70% in VHLS patients [4]. Patients who meet the VHLS diagnostic criteria are referred for molecular genetic testing. First, point mutations in the coding region and canonical splice sites of *VHL* are analyzed using next-generation sequencing (NGS) with a multigene panel or Sanger sequencing. If a negative result is obtained, the second step includes searching for large deletions in the region of localization of *VHL* using, for example, the MLPA (Multiplex Ligation-dependent Probe Amplification) method. The clinical specimen for molecular genetic testing is whole blood [5,6]. Patients with VHLS have one mutant and one normal allele; compound heterozygotes or homozygotes for some hypomorphic *VHL* alleles develop hereditary erythrocytosis not associated with the VHLS phenotype [7].

However, this recommended diagnostic algorithm has reduced analytical sensitivity for mosaic variants. Thus, a case study of a 55-year-old man has been published, who was hospitalized with metastatic CCRC (primary tumor in the left kidney, metastases in the lung) and pheochromocytoma of the right adrenal gland. Metastases in the liver and bones, as well as two hemangioblastomas in the spinal cord, were also found during the patient’s examination. The patient underwent a search for point mutations and copy number variation (CNV) in DNA from blood using an NGS panel containing 23 genes, including *VHL*. No pathogenic, likely pathogenic, or variants of uncertain clinical significance (VUS) were identified. However, DNA sequencing of the available primary tumors and metastases showed that all of them contained a likely pathogenic missense variant c.593T>C (p.Leu198Pro) in exon 3 of *VHL,* with a mutant allele fraction of 10–55%, which is unlikely for somatic mutations in primary tumors of different types from different organs. Re-analysis of the germline NGS data showed that this variant was also present in blood with a mutant allele fraction of 6%, but it was filtered out in the NGS data processing due to the minimum allele frequency (MAF) threshold being set at 10%, which is normal practice in NGS for germline variant search to cut off sequencing artifacts. Tumors harbored a higher content of cells with the *VHL* mutation than in the blood, as well as a deletion of the second *VHL* allele, so the mutation was detected in all tested samples. This case shows that if a standard diagnostic algorithm has failed to identify the cause of the disease in a patient who fully meets the clinical criteria for VHLS, then the search for the mutation should not be stopped [8]. Here, we describe a case of mosaic VHLS in a patient with hemangioblastomas and CCRC. He underwent molecular genetic testing using Sanger sequencing and MLPA twice with negative results, which did not allow us to recommend appropriate targeted therapy. Analysis of tumors allowed us to identify a recurring pathogenic variant, and NGS with high coverage for the mutant allele position showed the variant allele percentage as 2% in the blood; thus, VHLS was confirmed by laboratory methods in this patient.

## 2. Results

### 2.1. Absence of VHL Gene Alterations in Routine Testing

The patient fully met the VHLS diagnostic criteria: he had developed multiple CNS hemangioblastomas since the age of 12, a retinal tumor had been removed, and a CCRC resection was performed at the age of 32. However, the first molecular genetic study of *VHL* mutations in blood, performed in another laboratory, did not reveal any gene abnormalities. Considering the circumstances that the sequencing and annotation of variants was performed 14 years ago, and the other laboratory used self-designed probes for MLPA, we searched for *VHL* germline mutations again. We analyzed the *VHL* coding sequence with adjacent splice sites by Sanger sequencing and screened for large deletions by MLPA with MRC Holland probes. We also did not detect pathogenic/probably pathogenic variants and VUS in the *VHL* gene in the blood DNA of the patient.

### 2.2. VHL Mutation Analysis in Tumor Samples

**Figure 1:**
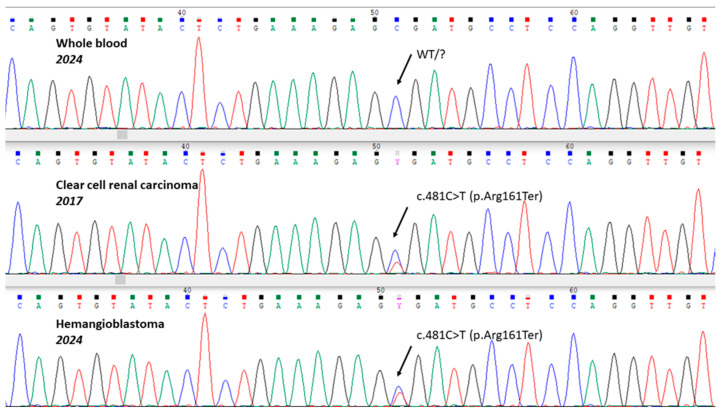
Variant c.481C>T (p.Arg161Ter) in different primary tumors of the patient; sequencing of *VHL* exon 3 with a reverse primer, WT—“wild type” (normal).

The second step of diagnostics consisted of searching for mutations in FFPE tumor samples. We had two blocks: one of CCRC from 2017 and one of hemangioblastoma from 2024. We detected the variant c.481C>T (p.Arg161Ter) in exon 3 of the *VHL* gene by the Sanger sequencing method in the DNA from both tumor samples with clearly visible mutant allele peaks, although with a different ratio of the variant/normal alleles (Figure 1). This substitution was classified as a pathogenic variant according to the ACMG recommendations and had previously been described in patients with VHLS. Since the occurrence of the same somatic mutation in primary tumors of different types and in different organs with interval of 7 years seems unlikely, an assumption was made about a possible mosaic form of VHLS in the patient. However, theoretically, the somatic nature of those mutations in tumors could not be completely excluded, and it was necessary to verify somatic mosaicism in DNA from the blood for the diagnosis of a congenital disease.

### 2.3. Deep High-Throughput Sequencing of the VHL Exon 3 in Blood

**Figure 2:**
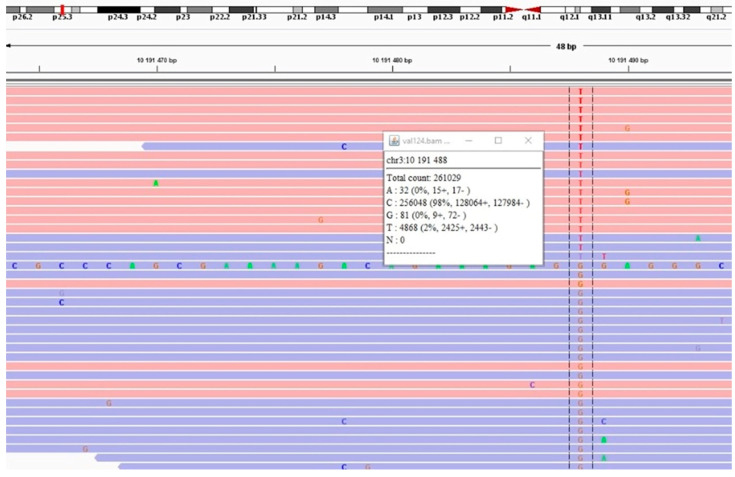
The position of the mosaic variant and its quantitative characteristics (proportion of the variant allele and number of forward and reverse reads) in IGV.

A blood genomic DNA sample, in which we had not previously detected *VHL* germline mutations using Sanger sequencing, was used to search for a mosaic variant. To obtain an amplification library, the Prep&Seq™ U-target module system was used, and primers were synthesized specifically for sequencing the region containing the position VHL:NM_000551.4:c.481C>T. Targeted sequencing on the Illumina NextSeq platform (“Illumina”, San Diego, CA, USA) allowed us to achieve coverage on the position of interest of x261029, which is sufficient to detect mosaicism (Figure 2). It was shown that 4868 reads demonstrated the variant allele T; the ratio of forward/reverse reads was 0.99 (2425/2443), other variant alleles at this position (A and G) were represented in less than 0.05% of reads, and one of them showed an 8-fold difference in the representation of forward/reverse reads, which allowed us to filter them out as sequencing artifacts. The proportion of the mutant T allele was determined to be 2%, which was further considered as molecular confirmation of the mosaic form of VHLS with low representation of the mutant allele. The predictive significance of the c.481C>T (p.Arg161Ter) mutation, according to the AMP/ASCO/CAP recommendations, was determined at the IA level; since the identified variant is the cause of the disease, in cases of multiple/metastatic tumors, targeted therapy by belzutifan is approved by the FDA and recommended in the NCCN guidelines [9,10]. It should be noted that we completed the confirmation of the mosaic form of VHLS and the preparation of the diagnostic report before the date of the concilium dedicated to targeted therapy options for the patient (previously, the recommendation of belzutifan had been postponed due to the lack of molecular genetic confirmation of the VHLS diagnosis).

## 3. Discussion

The importance of identifying mosaic forms of VHLS for the timely initiation of observation and genetic counseling became obvious in the early 2000s. Thus, two families were described in which children had hemangioblastomas, CCRC and/or pancreatic cysts, and germline *VHL* mutations. These germline mutations were not detected in their parents in peripheral blood by identical methods. However, in the first family, the proband’s mother had hemangioblastomas and pancreatic and renal cysts, and in the second family, the proband’s father also had CCRC, in addition to the three manifestations of the VHLS listed above. A more thorough study of DNA from leukocyte samples using FISH revealed a *VHL* deletion in the mother in the first family, and selective Sanger sequencing of an abnormal gel band detected a 3 bp *VHL* deletion in the father in the second family, corresponding to the mutations in the children. This result allowed us to differentiate the hereditary VHLS from the de novo case. The authors also demonstrated that the severity of clinical manifestations in mosaic VHLS was not less than that in the classical hereditary form of VHLS [11]. A case of a female patient was also described; she had adrenal pheochromocytomas that were removed at the ages of 11 and 18, and her father had a renal cyst and an epididymal cystadenoma due to *VHL* somatic mosaicism. Her father’s *VHL* mutation was also not detected by the conventional PCR and Sanger sequencing methods in the blood, and the authors applied high-performance liquid chromatography to detect PCR products with the mutation [12].

A case study with a high proportion of the mutant allele, which was missed during the first *VHL* mutation testing, was described later. The patient died due to progression of bilateral CCRC at the age of 66; she also had polycystic kidney and pancreas disease. Mosaicism was suspected when the mutation c.194C>G (p.Ser65Trp) was detected in the patient’s daughter, who had hemangioblastomas removed at the ages of 18 and 36. The fraction of the mutant allele in a repeated analysis of genomic DNA from blood was calculated as 19% of the *VHL* alleles in the patient, when PCR-RFLP was used and the intensity of staining of the mutant and normal bands in the gel was compared [13]. In 2000–2010, researchers used various custom modifications of different methods to detect the mutant *VHL* allele mixed with a large number of “wild-type” alleles without a unified approach. The analytical sensitivity of Sanger sequencing with the basic settings of a capillary genetic analyzer and programs for chromatogram analysis did not allow us to detect less than 10% of the mutant allele in the heterogeneous samples with an excess of the reference alleles [14].

The situation changed with the implementation of NGS into clinical practice, and it became possible to detect several percent of mutant alleles in a sample. According to various estimates, previously, up to 5% of patients had combinations of symptoms characteristic of VHLS, but they did not demonstrate a *VHL* germline mutation, which gave reason to suspect a mosaic form of this syndrome. Then, the proportion of somatic mosaicism in VHLS was estimated at 8.5% of cases using NGS [15]. The approach with multigene NGS panels has become common (to the point of surpassing Sanger sequencing [5]); it involves re-analysis of initially obtained sequencing data from blood DNA when the same mutation is detected in different tumors in a patient, or a second NGS test with high coverage of the region of interest—this approach results in the identification of a mosaicism with a 2% mutant *VHL* allele in the case described here. A similar case was described in a patient with hemangioblastomas that developed from the age of 11. Routine search for germline *VHL* mutations in the blood failed twice, with a large time interval between tests, and only NGS of the gene panel for the patient’s different tumors led to the diagnosis of the VHLS mosaic form [16]. Other examples of successful NGS testing could be provided: the search for mutations using NGS led to the diagnosis of the VHLS mosaic form in two patients, with percentages of the mutant allele of 1.7 and 5.7%, with the coverage of 4059× and 8902×, respectively. Moreover, there was a severe manifestation of VHLS in the form of bilateral CRCC and/or CNS hemangioblastomas (in the second patient since the age of 16) in the cases mentioned above [17]. Therefore, even if the analysis in a third-party laboratory did not detect a germline *VHL* mutation in a patient with characteristic features of VHLS, it is better to indicate in the medical documentation that a possible mosaic form of the disease cannot be excluded at this examination step (Table 1). An example is a report of a 38-year-old man who was diagnosed with cerebellar and spinal hemangioblastomas, CRCC, and polycystic pancreatic disease; however, NGS of a multigene panel using a standard pipeline for germline variants showed the absence of the *VHL* mutations on two occasions. Nevertheless, the authors reasonably recommended the analysis of FFPE samples to exclude the mosaic form of the disease [18].

**Figure 3:**
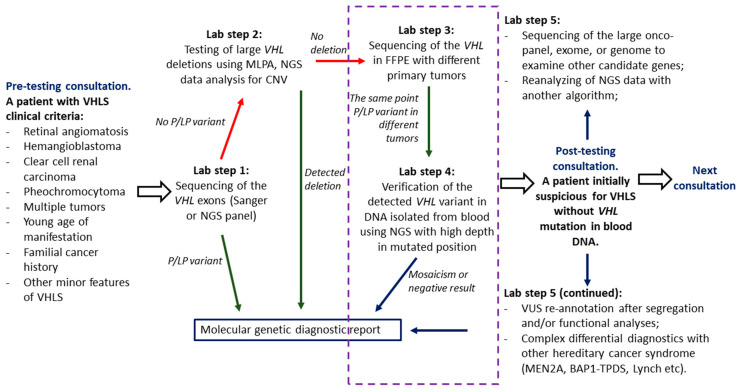
Flowchart of VHLS diagnostic algorithm with mosaicism evaluation. The mosaicism evaluation step is indicated by the dotted rectangle; VHLS—von Hippel–Lindau syndrome, NGS—next-generation sequencing, MLPA—Multiplex Ligation-dependent Probe Amplification, DNA—deoxyribonucleic acid, CNV—copy number variation, FFPE—formalin-fixed paraffin-embedded (blocks), P/LP—pathogenic/likely pathogenic, MEN2A—multiple endocrine neoplasia type 2A, BAP1-TPDS—BAP1 tumor predisposition syndrome.

In our opinion, if a patient has clinical features of VHLS, but sequencing and MLPA in routine testing did not identify any pathogenic or likely pathogenic *VHL* variant, diagnostics can be continued by the following (Figure 3):

- Analysis of *VHL* in tumors and subsequent re-analysis of DNA sequencing data from blood or a new NGS test with high coverage of the variant localization to detect the mosaic form of VHLS.
- Differential diagnostics with other cancer syndromes with overlapping phenotypes, such as VHLS type 2C—with MEN2A and hereditary pheochromocytoma/paraganglioma, or hereditary CCRC suspicious for VHLS—with BAP1-oncosyndrome and inherited balanced translocation t(3;8) [1,2,19].
- Validation of VUS by segregation and/or functional analysis, especially for potential splice site mutations.
- Using higher-throughput techniques, including whole-genome sequencing (WGS), which can sometimes give unexpected results. For example, the patient has been developing CNS hemangioblastomas and multiple CCRCs since the age of 20 and has no family history of VHLS; testing for point germline mutations and extended *VHL* deletions was negative. WGS detected a de novo balanced translocation t(1;3)(p36.3;p25) with a breakpoint in the intron 2 in the *VHL*, which could be considered as an inactivating event for the altered allele and a confirmation of the VHLS diagnosis [20]. Another patient, also without a family history of VHLS, has been treated for retinal angiomatosis, CNS hemangioblastomas, and multiple CCRCs since the age of 37. Sanger sequencing and MLPA did not obtain any germline *VHL* mutations. The WGS trio showed that the patient had a de novo germline variant, c.236A>G (p.Tyr79Cys), in a heterozygous state in the *ELOC* gene. This gene encodes elongin C—the main partner of pVHL in a complex for hypoxia-inducible factor (HIF) degradation. The authors further demonstrated that this variant at the functional level leads to consequences similar to inactivating *VHL* mutations [21]. It is noteworthy that the identical somatic variant is the most common substitution determining the “associated with the *ELOC* mutation type of renal carcinoma” according to the new WHO 2022 classification of kidney tumors [22]. In other words, VHLS has acquired a probable second minor candidate gene.

Comparison of the sensitivity for mosaic variant detection using Sanger sequencing and NGS is complicated as it is dependent upon many factors. The Sanger sequencing result is affected by the basic settings of the capillary sequencer program for the artifact cutoff line, the signal intensity from the labeled fragments, the presence of minor signal noise due to ineffective dideoxyterminator removal or non-specific PCR products, and the experience of the laboratory geneticist who analyzes the chromatogram. In turn, the sensitivity of the NGS approach depends on the depth of sequencing at the mutant position, MAF, the laboratory pipeline, and the availability of the patient’s phenotypic data for the identified variant annotation. In general, a mosaic variant percentage below 10% of the *VHL* alleles is more prone to false-negative results [8,13,14,15,16,17]. Similar mosaic cases of other hereditary cancer syndromes also demonstrate critical levels of mosaicism, with a mutant allele proportion of 0–10%, requiring high-depth NGS/digital PCR for variant verification [23]. For example, a patient with colorectal and endometrioid cancers and mosaic Lynch syndrome was described. She had an *MSH6* pathogenic mutation with a 5.34% allele frequency in normal colonic tissue and 1.64% in blood DNA [24]. A study of patients with tuberous sclerosis also demonstrates a critical level of 10% for variant allele frequency in mosaicism detection in blood by conventional methods; the average fraction of mutant alleles in such low-level mosaic patients was 6.8% [25].

Establishing the diagnosis of VHLS is important for determining surgical treatment tactics. Unlike sporadic CCRC (this tumor is removed after diagnosis), VHLS patients need to preserve the functioning renal parenchyma as long as possible due to bilateral and/or multiple CCRCs. Therefore, the “3 cm rule” is used: the tumor is removed when it reaches 2.5–3 cm in the largest dimension by small resection, if this tumor does not invade the renal capsule, calyces/pelvis, or large vessels [26,27].

Identification of VHLS mosaic cases is also important because it allows us to treat patients with belzutifan—a selective HIF2α inhibitor. An increasing concentration of HIF in the cell is a consequence of the absence of a functionally active pVHL and is considered as the main pathogenic event during carcinogenesis in VHLS-associated tumors [28]. Belzutifan is an FDA-approved drug for the systemic therapy of CCRC, hemangioblastomas, and pancreatic neuroendocrine tumors that do not require immediate surgery in patients with a confirmed diagnosis of VHLS. This option may be the only reasonable targeted therapy in VHLS in some cases, for example, for patients with multiple progressing hemangioblastomas, because other kinase inhibitors will be less effective [9,29,30]. Computer drug design and artificial intelligence tools (AlphaFold) may facilitate the prognosis and development of personalized targeted therapy for VHLS in the future, considering the type of mutation and its expected functional consequences in an individual patient [31,32].

A diagnosis of VHLS confirmed by the molecular genetic method provides an opportunity for the patient to receive active surveillance in accordance with the recommendations of professional medical associations aimed at early tumor detection. The first special examinations are recommended starting in early childhood, which are described in the recommendations of 2017 [33]. The VHL Alliance recommendations of 2020 are currently widely used. In accordance with these recommendations, the following diagnostic examinations are carried out yearly:

- Physical and dilated eye examination from age 1 year (retinal angiomatosis);
- Blood pressure and pulse measurement from age 2 years, and metanephrine analyses from age 5 years (pheochromocytoma);
- MRI of the brain and spine with/without contrast, and audiogram from age 11 years every 2 years (hemangioblastoma);
- MRI of the internal auditory canal (once) and abdomen from age 15 years every 2 years (CCRC, pheochromocytoma, renal and pancreatic lesions).

A number of specific active surveillance tests stop at age 65 years. If hemangioblastomas are present and there is an increase in size, or if the patient has associated symptoms, scans should be conducted yearly (or more frequently, not every 2 years), as appropriate (or referred to neurosurgery). Similarly, if small renal tumors (<3 cm) are found, reimaging with MRI should be conducted every 3–6 months to determine stability. Once stability has been determined over three consecutive scans, consider extending the frequency to every 2 years. If the renal mass is >3 cm, consider a referral to a urologist (preferably familiar with the care of VHLS) [34]. The start of the screening procedure may vary slightly from the VHL Alliance document in more recent recommendations. For example, the Danish consortium recommendations of 2022 established starting MRI of CNS for hemangioblastoma detection at age 10 [5], while the study in Germany in 2024 provided a rationale to start MRI scans for CNS hemangioblastomas at age 12 [35]. These data demonstrate the importance of the *VHL* mutation identification for active surveillance, therapy options, and the type of surgical intervention decisions in patients with VHLS.

## 4. Materials and Methods

**Patient’s anamnesis.** A 39-year-old man was admitted to the Research Centre for Medical Genetics in 2024 for medical genetic counseling and molecular genetic diagnostics regarding VHLS. He had no familial cancer history. The first hemangioblastomas were detected in the patient at the age of 12, at which time enucleation and prosthetics of the left eye were performed. Then, stereotactic radiotherapy with hypofractionation was performed on hemangioblastomas in the region of the 4th cervical (3.3 cm^3^) and 9th thoracic (2.8 cm^3^) vertebrae in 2010. Molecular genetic analysis by Sanger sequencing and MLPA performed in a third-party laboratory in 2010 did not detect a *VHL* mutation. Radiosurgical treatment of hemangioblastomas in the medulla oblongata and basal parts of the cerebellum, along with stereotactic management of the tumors at the level of C7-Th1, was performed in 2013, and the removal of cerebellar hemangioblastoma and a node at the level of C3-4 was performed in 2014–2015. Stereotactic surgical treatment of 6 small cerebellar hemangioblastomas (from 0.03 to 0.6 cm^3^) was performed in 2017, and tumors at the levels of Th7 and Th11 were treated in 2018. A CCRC measuring 4 × 2.5 × 2.7 cm was surgically removed in 2017 by a partial resection of the right kidney. Since 2020, new intracranial foci have appeared, and tumors in the cervical spine have increased (at the thoracic and lumbosacral levels—hemangioblastomas without significant dynamics). The CyberKnife (“Accuray”, Madison, WI, USA) was used for the radiosurgical treatment of 5 hemangioblastomas in the left cerebellar hemisphere, followed by treatment of foci at the C5-Th1 level in 2021. The patient had unfavorable symptoms in 2022: progressive weakness of the lower extremities and a severe pain syndrome. An intramedullary hemangioblastoma was detected at the Th9 level with hemorrhage in the spinal cord; the tumor was removed microsurgically under neurophysiological monitoring (pathology report: spinal cord hemangioblastoma, grade 1); then, the patient received rehabilitation treatment. Since early 2024, negative dynamics have been noted in the development of CNS hemangioblastomas, and systemic targeted therapy has been considered.

**DNA isolation.** Genomic DNA was isolated from whole blood using the GentaMag Blood HMW DNA Kit (“Genterra”, Moscow, Russia). Formalin-fixed paraffin-embedded (FFPE) tumor samples were processed by the RecoverAll Total Nucleic Acid Isolation Kit (“ThermoFisherScientific”, Waltham, MA, USA) for tumor DNA isolation. The DNA concentration was measured using a Qubit 3.0 fluorimeter (ThermoFisherScientific, USA).

**Polymerase chain reaction (PCR).** PCR was performed in a C1000 thermocycler (“BioRad”, Hercules, CA, USA). A single reaction contains 2.5 μL of 10× PCR buffer (“SibEnzyme”, Novosibirsk, Russia), 1.5 μL of 25 mM MgCl_2_, 2 μL of dNTPs (2.5 mM each), 5 M of forward and reverse primers, 2.5 U of Taq polymerase, 1 μL of DNA, and up to 25 μL of deionized water. The primer sequences for the amplification of *VHL* exons 1–3 were identical to those we used previously [36]. Temperature parameters: initial denaturation at 95 °C for 2.5 min, then 38 cycles (95 °C 45 s, 61 °C 30 s, 72 °C 30 s), and final elongation at 72 °C for 2 min.

**Sanger sequencing.** PCR products were treated with exonuclease I from *E. coli* (2 U; Fermentas, Lithuania) and alkaline phosphatase (1 U; SibEnzyme, Russia) to remove unreacted primers and dNTPs. A 2.5 µL aliquot of the processed PCR product was used as a template in a Sanger sequencing reaction using the BigDye^®^ Terminator v3.1 Cycle Sequencing Kit (“ThermoFisherScientific”, Waltham, MA, USA) according to the manufacturer’s instructions. Detection of labeled fragments was performed on a 3500 Capillary Genetic Analyzer (“ThermoFisherScientific”, Waltham, MA, USA). Sequencing chromatograms were analyzed with Chromas v2.6.6 software (“Technelysium”, Brisbane, Australia), compared with the *VHL* reference sequence (NM_000551.4) using the Ensembl genome browser (https://www.ensembl.org/ (accessed on 24 May 2024)).

**Variant annotation.** The pathogenicity of germline variants was determined according to the ACMG/AMP guidelines [37] using the ClinVar (https://www.ncbi.nlm.nih.gov/clinvar/ (accessed on 18 July 2024)), LOVD (https://www.lovd.nl/ (accessed on 18 July 2024)), Franklin (https://franklin.genoox.com/clinical-db/home (accessed on 18 July 2024)), and Varsome (https://varsome.com/ (accessed on 18 July 2024)) databases. The predictive (therapeutic) value of genetic variants was assessed according to the AMP/ASCO/CAP criteria [38] using the COSMIC (https://cancer.sanger.ac.uk/cosmic (accessed on 18 July 2024)), Franklin, Varsome, and MyCancerGenome (https://www.mycancergenome.org/ (accessed on 18 July 2024)) databases.

**Multiplex Ligation-dependent Probe Amplification (MLPA).** *VHL* deletion analysis was performed by MLPA using the SALSA MLPA P016 VHL kit (“MRC Holland”, Amsterdam, The Netherlands), which includes 17 probes in the *VHL* localization region: 9 intraexonic, 6 gene-flanking, and 2 pericentromeric/telomeric probes on 3p, as well as 12 reference probes for other genomic regions not involved in VHLS. Fragment analysis and CNV evaluation were performed using a 3500 Genetic Analyzer (ThermoFisherScientific, USA) and Coffalyser software (https://www.mrcholland.com/technology/software/coffalyser-net (accessed on 25 January 2025) MRC Holland, The Netherlands); the heterozygous deletion interval was set at 0.40 < FR < 0.65.

**High-throughput sequencing.** Verification of the nucleotide sequence variant with low representation of the alternative allele was carried out using the NGS method with an increased reading depth of the target region. The library was prepared using the Prep&Seq U-target DNA primer design basic module (“Parseq Lab”, Saint-Petersburg, Russia) according to the manufacturer’s protocol. Synthesis of uracil-modified primers F:TCCTTGTACUGAGACCCUAGUC and R:ACATTTGGGUGGUCTUCCAG was performed by the “Syntol” company, Moscow, Russia. Sequencing was performed on an Illumina NextSeq500 (“Illumina”, San Diego, CA, USA) using the paired-end reading method (2 × 150 bp). Sequencing data were processed using an automated algorithm that included alignment of reads to the reference genome sequence (GRCh37/hg19) and postprocessing. Functional significance and filtration of known neutral variants were analyzed using the gnomAD database v.4.1 and ANNOVAR v.4.7 [39], and manual filtering of sequencing artifacts and analysis were performed using Integrative Genomic Viewer (IGV) v.2.4.14 [40]. Genetic variants were annotated in accordance with the HGVS nomenclature (http://www.hgvs.org/mutnomen (accessed on 30 September 2024)).

## 5. Conclusions

In conclusion, we would like to highlight several important points:

- Incidence of VHLS and the mosaic form of this disease may be higher than was previously thought;
- Severity of the clinical symptoms and age of manifestation do not differ among mosaic and “classic” heterozygotes for *VHL* mutations;
- Timely diagnosis of VHLS (including its mosaic form) is important for monitoring mutation carriers, determining surgical treatment options, and administering targeted therapy with belzutifan;
- Detection of mosaic VHLS by analyzing blood DNA can now be effectively performed using NGS, especially if FFPE tumor samples from patient are available.

Thus, timely detection of mutations and confirmation of diagnosis directly influence decisions concerning active surveillance, options of surgical treatment, and targeted therapy in patients with VHLS.

## Figures and Tables

**Table 1:** False-negative results in routine VHLS testing due to mosaicism.

Patient’s Phenotype	Routine Method–Negative Result	Mosaicism Verification	Reference
Case #1: HBs, pancreatic and renal cysts; Case #2: CCRC, HBs, pancreatic and renal cysts.	SS	Case #1: FISH; Case #2: SSCP > SS of the abnormal band	[11]
Renal cyst, epididymal cystadenoma	SS	HPLC	[12]
Bilateral CCRC, renal and pancreas cysts	SS	PCR-RFLP when point mutation was detected in his daughter	[13]
HBs	SS (twice)	NGS panel for primary tumors and blood DNA	[16]
Case #1: HB, pheochromocytoma, pancreatic endocrine tumor; Case #2: bilateral CCRC	SS	NGS panel for the *VHL* gene only	[17]
HBs, CCRC, pheochromocytoma	SS and NGS panel with MAF 10%	NGS panel for primary tumors > re-analysis of NGS data with low MAF for blood sample	[8]
HBs, CCRC, retinal angiomatosis	SS (twice), MLPA	SS of primary tumors DNA > NGS panel for targeted locus	this case report

HB—hemangioblastoma, CCRC—clear cell renal carcinoma, SS—Sanger sequencing, HPLC—high-performance liquid chromatography, SSCP—single-strand conformation polymorphism, PCR-RFLP—polymerase chain reaction–restriction fragment length polymorphism, MAF—minor allele frequency, DNA—deoxyribonucleic acid, NGS—next-generation sequencing, MLPA—multiplex ligation-dependent probe amplification.

## Data Availability

The data presented in this study are available on request from the corresponding author (the data are not publicly available due to privacy and legal restrictions).

## Abbreviations

The following abbreviations are used in this manuscript:

VHLS	von Hippel–Lindau Syndrome
CCRC	Clear Cell Renal Carcinoma
MLPA	Multiplex Ligation-dependent Probe Amplification
NCCN	National Comprehensive Cancer Network
ACMG	American College of Medical Genetics
ASCO	American Society of Clinical Oncology
FFPE	Formalin-Fixed Paraffin-Embedded
HGVS	Human Genome Variation Society
COSMIC	Catalogue Of Somatic Mutations In Cancer
MEN2A	Multiple Endocrine Neoplasia type 2A
DNA	Deoxyribonucleic Acid
IGV	Integrative Genomic Viewer
CNS	Central Nervous System
NGS	Next-Generation Sequencing
CNV	Copy Number Variation
MAF	Minor Allele Frequency
VUS	Variant of Uncertain Significance
PCR	Polymerase Chain Reaction
PCR-RFLP	Polymerase Chain Reaction–Restriction Fragment Length Polymorphism
AMP	Association for Molecular Pathology
USA	United States of America
FDA	Food and Drug Administration
CAP	College of American Pathologists
HIF	Hypoxia-Inducible Factor
WGS	Whole-Genome Sequencing
WHO	World Health Organization
MRI	Magnetic Resonance Imaging
